# Relationships between Interpersonal Goals and Loneliness in Older Adults: A Cross-Sectional Study

**DOI:** 10.3390/ijerph20031914

**Published:** 2023-01-20

**Authors:** Francesca C. Ezeokonkwo, Kathleen L. Sekula, Jeffrey E. Stokes, Laurie A. Theeke, Rick Zoucha, Meredith Troutman-Jordan, Dinesh Sharma

**Affiliations:** 1 School of Nursing, James Madison University, Harrisonburg, VA 22807, USA; 2 School of Nursing, Duquesne University, Pittsburgh, PA 15282, USA; 3 Department of Gerontology, McCormack Graduate School of Policy and Global Studies, University of Massachusetts, Boston, MA 02125, USA; 4 School of Nursing, George Washington University, Washington, DC 20006, USA; 5 School of Nursing, University of North Carolina Charlotte, Charlotte, NC 28223, USA; 6 Department of Mathematics and Statistics, James Madison University, Harrisonburg, VA 22807, USA

**Keywords:** loneliness, interpersonal goals, compassionate and self-image goals, older adults

## Abstract

Loneliness is linked to many physiological and psychological issues and disproportionately affects older adults. Interpersonal goals (compassion and self-image) are essential to interpersonal relationships; however, how they relate to loneliness in older adults is unknown. We investigated the impact of interpersonal goals on loneliness using the Ecosystem–Egosystem Theory of Social Motivation. This study, adopting a descriptive cross-sectional correlational design, used data from the 2016 Health and Retirement Study. Participants (*n* = 3212) included people aged >65 years (mean age: 75; female: 60.1%). We performed exploratory factor analysis with principal axis factoring and varimax rotation to examine the suitability of compassionate and self-image goals as separate factors. The complex samples general linear model was used to assess the relationship between loneliness and interpersonal goals. Interpersonal goals were significantly negatively associated with loneliness. Respondents with higher compassion and self-image goals reported lower loneliness levels. Our results contribute to understanding how interpersonal goals relate to loneliness in older adults. These initial findings warrant further investigation.

## 1. Introduction

Loneliness—operationally defined as a negative subjective experience resulting from discrepancies between individuals’ desired and perceived number and closeness or quality of social relationships—is conceptualized as a psychological state simultaneously constituting a yearning for human contact as well as a feeling of aloneness [1]. Although loneliness affects people across all developmental stages, older adults are at great risk of loneliness [2] due to physical health deterioration and the loss of family and friends [3,4], as well as a lack of social resources to initiate new relationships to compensate for such losses, particularly among the oldest old [4].

Loneliness among older adults substantially impacts their quality of life [5,6]. Compared to those not experiencing loneliness, older adults exhibiting chronic loneliness report less exercise, greater tobacco use, a greater number and severity of chronic illnesses, higher depression levels, and a greater average number of nursing home stays [7].

Loneliness precipitates diminished sleep quality, shorter sleep duration, lower sleep efficiency, greater daytime fatigue in later adulthood, and reduced subjective sleep quality [8]. A 2022 study by Bogart et al. examined the cross-sectional associations between loneliness and inflammatory markers among older adults and found that higher trait loneliness and aggregated momentary measures of loneliness were associated with higher levels of C-reactive protein (CRP) [9].

The incidence of loneliness among U.S. older adults varies across studies and has been estimated to be as high as 60% in older frail adults [10]. Theeke [5] documented that 19.3% of community-dwelling older adults reported feeling lonely. In a 2015 survey of older Americans, nearly 55% of the study sample reported feeling some level of loneliness, with 27% reporting moderate and 28% reporting severe loneliness [11]. Perissinotto et al. [12] documented a 30–43% prevalence of loneliness among older community adults, whereas data from a survey by the American Association of Retired Persons (AARP) estimated that 25% of community-dwelling U.S. respondents over the age of 70 years were lonely [13].

Previous research has documented the following loneliness predictors in older adults: female sex, living alone, low income, low economic status, and age (older than 65 [14]). Older adults are at increased risk for loneliness due to physical health deterioration, retirement, relocation, and loss of family and friends through death or separation [2,3,15]. In older adults, studies have demonstrated that loneliness elicits both physiological and emotional stress responses that are linked to morbidity and mortality [16,17,18]. It is critical to understand the influence of loneliness on health and aging as it is known that lonely older people do utilize healthcare resources more frequently [19].

As loneliness—although an individual emotional experience—is inherently linked with one’s social context and relationships, the importance of adults’ own social desires and goals should be considered when assessing loneliness in later life. Foremost among these are interpersonal goals, which include compassion and self-image goals—the primary constructs of the Ecosystem–Egosystem Theory of Social Motivation. Compassionate goals involve focusing on supporting others rather than personal self-gain with the intention of facilitating others’ well-being. Self-image goals involve constructing, maintaining, and defending a desired public or private image of the self to pursue one’s own interests [20]. These contrasting social goals represent distinct motivational perspectives on the relationship between the self and others and have strong implications for promoting or undermining interpersonal relationships, respectively [21,22]. Compassionate goals relate to feelings of clarity, connectedness, and closeness to others, fewer interpersonal conflicts, and high positive emotions, thereby mitigating feelings of loneliness. By contrast, self-image goals relate to feelings of fear and confusion, greater loneliness, interpersonal conflicts, and low positive emotions [22]. Compassionate goals foster social support and trust, while self-image goals undermine them [20]. Interpersonal goals may offer a new perspective to examine loneliness in older adults.

Numerous studies have elucidated the prevalence of loneliness among older adults [15,23]. Studies have also explored the effect of interpersonal goals on loneliness in young populations [22,24]. However, research has not yet established the relationship between interpersonal goals and loneliness among older adults. This is an important gap in current literature, not only because loneliness increases in later life but also because older adults exhibit distinct social and emotional goals in comparison with younger and midlife adults [25]. Our study fills this knowledge gap by examining the direct relationship between loneliness and compassion and self-image goals in older adults. Understanding how interpersonal goals relate to loneliness might aid healthcare providers in developing targeted interventions that mitigate loneliness in older adults.

### Theoretical Framework

This study was guided by the Ecosystem–Egosystem Theory of Social Motivation [20]. Ecosystem motivation promotes close and mutually supportive relationships through behaviors that are intended to be constructive and supportive. People with an ecosystem motivational perspective perceive others as connected with them, show concern about others’ well-being, and treat their own and others’ needs and desires equally, with an understanding that they are part of a larger whole. People with ecosystem motivation tend to adopt compassionate goals [26]. Egosystem motivation focuses on proving and validating self-worth. demonstrating desired qualities, and involves concerns regarding others’ impressions, thereby precipitating self-consciousness and social anxiety [26]. People with an egosystem motivational perspective show greater concern regarding the fulfillment of their own needs and desires but fail to exhibit concern for others’ well-being. They perceive the relationship between the self and the other as competitive; therefore, they do not regard others’ needs and desires as equally important. People with egosystem motivation primarily focus on themselves and adopt self-image goals, which may diminish the social support received from others and, therefore, result in loneliness [20,26].

As hypothesized by the ecosystem–egosystem theoretical framework, compassionate and self-image goals reflect distinct ways of thinking or perspectives. Altruistic motivation (for others’ benefit) and egoistic motivation (for self-benefit) represent contrasting goals and feelings. People for whom others’ well-being is genuinely significant adopt compassionate goals and, consequently, exhibit a positive affect, a sense of clarity, and interpersonal closeness. When people aim to benefit or protect themselves and consider the relationship between themselves and others an egosystem, they adopt self-image goals and, consequently, exhibit fearful feelings, confusion, and interpersonal conflict [18]. Evidently, compassionate and self-image goals prevail at opposite ends. However, people occasionally exhibit overlaps between these two perspectives for short periods [27]. People exhibit self-image goals from an ecosystem perspective and compassionate goals from an egosystem perspective. Distress and interpersonal goals are mutually reinforcing–greater distress may discourage compassionate goals and encourage self-image goals [20].

This study’s objective was to investigate interpersonal goals’ (compassionate and self-image goals) impact on loneliness in older adults.

This study incorporated a descriptive cross-sectional, correlational secondary data analysis. The primary research question in this regard was, “How is loneliness in older adults associated with interpersonal goals (compassionate and self-image)?” Loneliness was the outcome variable, whereas compassionate goals and self-image goals were the predictor variables. Note that where mentioned below, “loneliness” refers to “loneliness in adults”. The two hypotheses of the study were as follows:

**Hypothesis** **1 (H1).** *Compassionate goals will be associated with lower loneliness*.

**Hypothesis** **2 (H2).** *Self-image goals will be associated with greater loneliness.*

## 2. Materials and Methods

Study data were derived from the following two components of the Health and Retirement Study (HRS) data file (publicly available data): the 2016 HRS core dataset of the public biennial survey data (https://hrs.isr.umich.edu/about (accessed on 6 November 2020)), the RAND, and the Psychosocial and Lifestyle Questionnaire. As this study applied secondary data analysis using de-identified data, IRB approval was not required. Our analyses used data from 2016 when interpersonal goal measures were introduced in the HRS survey. The response rate for the HRS is high—typically 85–90% [28], and somewhat lower for the Psychosocial and Lifestyle Questionnaire—generally 73–88% [29]. Inclusion criteria were community-dwelling older adults aged 65 years and older who completed the HRS survey and Psychosocial and Lifestyle Questionnaire without needing a proxy. In 2016, the HRS included 20,912 participants. The following respondents were excluded: individuals under the age of 65 (*n* = 10,940), participants living in the nursing home (*n* = 429), and those who completed the survey by proxy (*n* = 450). Further, because HRS administers the Leave Behind Questionnaire (LBQ) to a random 50% subset of HRS households at alternating waves, we also excluded participants who were either not eligible for the LBQ in 2016 (*n* = 4683) or did not complete it (*n* = 998). Of the 3412 participants fulfilling the inclusion criteria, 200 (6%) respondents with missing values for items corresponding to the research variables were excluded. Thus, the final sample comprised 3212 cases.

A power analysis was conducted using the G*Power 3.1 software [30], which indicated that 159 participants were required to obtain a medium effect size of f = 0.25—with standard power and standard alpha of 0.80 and 0.05, respectively.

The revised 11-item UCLA Loneliness Scale [31] was used by the HRS to measure participants’ loneliness within the past week. After reverse-coding four negatively worded items, the overall loneliness score was computed as the 11 items’ average. Higher scores indicated greater loneliness. The internal reliability of the 11-item UCLA exhibits a Cronbach’s α value of 0.87 [31].

A modified six-item measure of interpersonal goals was used to assess compassionate and self-image goals [20,32]. Three items assessed compassionate goals: “compassion for others”, “supportive of others”, and “avoid being selfish”. Three items assessed self-image goals: “get others to see your positive qualities”, “get others to respect you”, and “avoid appearing unattractive”. The six items were reverse-coded, and the scores ranged from 1 (not at all) to 5 (extremely) [29]. In the original study of the 13-item scale, Cronbach’s alpha was 0.90 for compassionate goals and 0.83 for self-image goals [24].

We computed Cronbach’s alpha reliability coefficients for the scales of loneliness, compassionate goals, and self-image goals. For this current study, reliability for loneliness was high (α = 0.87), which was the same as documented by previous research [31], but lower for compassionate goals (α = 0.68) and self-image goals (α = 0.60). We also performed exploratory factor analysis to examine the suitability of compassionate and self-image goals as separate factors [20,29,32]. In this analysis, two factors were forced with principal axis factoring as the extraction method and a varimax rotation. Table 1 displays the rotated factor loadings. All compassionate goal items loaded strongly (>0.52) on Factor 1 and weakly (<0.40) on Factor 2, supporting compassionate goals as a distinct factor. Two items of self-image goals loaded strongly (>0.61) on Factor 2 and weakly (<0.40) on Factor 1. The remaining item of self-image goals (avoid appearing unattractive) did not load strongly on either factor (<0.40), potentially indicating a difference in the salience of “appearing unattractive, unlovable, or undesirable to others” among older vs. younger adults [25]. Conforming to both prior research using these scales [20,32] and HRS guidelines for variable construction [29], the “appearing unattractive” item was retained in the self-image goals scale used in the present analyses.

Further, we included sociodemographic (age, sex, race, ethnicity, functional impairment, homecare utilization) and socioeconomic (education, income, employment status, household size) covariates based on a review of prior literature. The reference categories selected included male sex, an educational level lower than high school, retired or not in the labor force for employment status, no functional impairment, and home care utilization.

We performed linear regressions using the complex samples general linear model command in SPSS. The complex samples analysis procedure used the PLBWGTR variable as the sampling weight, the SECU variable as the primary sampling unit, and the STRATUM variable as the sampling strata [33,34]. We computed three sets of complex sample linear regression: (1) regression with compassionate goals predicting loneliness, (2) self-image goals predicting loneliness, and (3) the full model with compassionate and self-image predicting loneliness. In all three sets of analyses, we controlled for the aforementioned sociodemographic and socioeconomic variables.

## 3. Results

Table 2 presents the descriptive statistics. Participant ages ranged from 65 to 99 years (mean 75.7), predominantly in the 65–74 years age group (44.9%); Female (60.1%); White (81.0%), non-Hispanic (90.6%); the most common level of education among participants was high school graduate (31.0%); and 80.0% were retired or not in the labor force. Most participants (89.6%) had not utilized home care in the previous two years; 83.5% exhibited no functional impairment. On average, participants’ income was $14,458.34, and the number of people in the household was two.

Hypothesis 1: As hypothesized, compassionate goals were a significant negative predictor in both the separate model (B = −0.16, *p* < 0.001) and the combined model (B = −0.14, *p* < 0.001). This result indicates that those with a higher level of compassionate goals exhibited lower loneliness after controlling for other factors (Table 3 and Table 4).

Hypothesis 2: We found that self-image goals were a significant negative predictor in the separate model (B = −0.09, *p* < 0.001) and combined model (B = −0.03, *p* = 0.015), indicating that those with higher levels of self-image goals exhibited lower loneliness after controlling for other factors (Table 4 and Table 5). Thus, our second hypothesis was not supported.

Table 4 displays the parameter estimates for the regression with compassionate goals, self-image goals, and the control variables predicting loneliness. Compassionate goals (B = −0.14, *p* < 0.001) and self-image goals (B = −0.03, *p* = 0.015) were significant negative predictors. Having a college or higher degree, functional impairment, and healthcare utilization all correlate with loneliness (Table 3, Table 4 and Table 5).

## 4. Discussion

This secondary data analysis is the first study to explore the relationship between interpersonal goals and loneliness among older adults. To the best of our knowledge, these factors have not been previously examined in relation to loneliness in older adults. The research findings demonstrate that loneliness decreases as interpersonal compassion and self-image goals increase. This study is rooted in the study by Crocker and Canevello [20], which introduced interpersonal goals through their Ecosystem–Egosystem Theory of Social Motivation.

Per this study’s results, one hypothesis was supported and one was not. The first hypothesis was confirmed, indicating that loneliness was lower among those with greater compassionate goals. These findings are consistent with prior studies’ findings [20,22,24]. In previous studies, having more compassionate goals has also been linked to decreased symptoms of anxiety and depression [24], increased self-esteem [35], feeling more peaceful and less isolated [34], increased constructive approaches to interpersonal problems [36], and increased satisfaction with life [37].

The second hypothesis, that is, self-image goals would be associated with greater loneliness, was not supported. Indeed, we found a significant *negative* association between self-image goals and loneliness, contradicting previous studies: As self-image goals increase, loneliness increases among college students [20,22,24]. As the comparative study participants were from a younger population, these contradictions could be because of the different priorities of college students and older adults. In particular, as older adults age, they become less focused on fostering larger social networks or initiating new social relationships and instead focus more heavily on their closest and most rewarding relationships [25]. Therefore, self-image goals may provoke loneliness among younger adults aiming to impress new people and grow their social networks but play an entirely different role among an older population seeking *quality* rather than *quantity* in their social relationships.

No definitive data explain the difference between younger and older adults regarding self-image goals. However, consistent with Erikson’s psychological development stages [38], college students are preoccupied with the self-image-driven, egocentric, and competitive establishment of role/career identity and intimate partnerships—based on a perceived fear of scarcity and inexperience with the value of collaborative effort. However, older adults, who may exhibit relatively greater satisfaction with their lifelong accomplishments, would have arrived at an appreciation of the importance of collective—and perhaps growing—dependency on cooperative effort, thus adopting a more altruistic and compassionate perspective. Consequently, we determined that differentiating between young and older adults is important.

In the full linear regression model, higher scores on both compassionate and self-image goals were significantly related to decreased loneliness. Participants with higher levels of compassion and self-image goals reported lower loneliness levels. Previous studies have found that females and older individuals are more likely to experience elevated loneliness [39,40,41]. However, like the findings of the HRS secondary analysis by Theeke [5], the meta-analysis by Maes et al. [42], and another study by Sunwoo [43], this study found no significant sex- and age-related differences in loneliness. As documented previously [44,45], our study found a strong positive correlation between functional impairment and loneliness. A lower educational level is also correlated with greater loneliness [45]. Similar to Theeke [5], our study found no association between the frequency of home care utilization and loneliness. Further, race, ethnicity, income, household size, and employment status were not significant loneliness predictors.

A key strength of this study is the use of a large population-based, nationally representative survey. This study can be replicated using international-level data to provide a meaningful comparison from a multicultural perspective. The study focused on interpersonal goals and advanced frontiers for researchers to further explore how interpersonal goals relate to loneliness in older adults. Finally, this study’s results contribute to the growing body of knowledge regarding loneliness in older adults and can act as a valuable reference for examining post-COVID-19 loneliness in older adults.

This study exhibited some limitations. Data were obtained from a secondary source; therefore, only the variables available in the dataset were used. Second, verifying cause and effect was impossible owing to the study’s cross-sectional nature. Third, the study primarily focused on providing a broad overview of interpersonal goals’ effect on loneliness among older adults; thus, additional longitudinal and experimental research is required to inform our understanding in this area. While most findings were consistent with those of previous studies, this was the first exploration of interpersonal goals’ effect on older adults; therefore, these results cannot be compared to those of previous studies. Fourth, Blacks and Hispanics were underrepresented in the study sample. Thus, the results cannot be generalized beyond the races and ethnicities included. Finally, the HRS survey comprises self-reported data, which are subject to response bias that may directly or indirectly influence the study’s outcomes.

Despite the study’s limitations, our findings are meaningful and provide a satisfactory foundation for future research. This study advances our understanding of the benefits of genuinely caring for—and extending support to—others. Further, this study opens novel avenues for the development of psychological interventions to mitigate loneliness. Disseminating these findings may aid public health policymakers and healthcare workers. The role of interpersonal goals in loneliness among older adults is under-researched.

This study’s results have implications for future research and practice. In the present study, individuals’ compassion and self-image goals were both associated with reduced loneliness among older adults. First, these results underline the differences between older and younger adults concerning their social goals and behaviors. Second, the findings elucidate the importance of identifying personal perspectives or resources that may be targeted in interventions aimed at reducing loneliness and preventing its adverse effects on the health and longevity of older adults.

In particular, healthcare providers play a pivotal role in assessing and recognizing loneliness and ensuring that patients receive appropriate care and treatment. Therefore, healthcare practitioners should assess loneliness in older adults; this is especially important for community and home healthcare practitioners providing home care to older adults. Healthcare providers should particularly focus on and conduct extensive loneliness assessments using standardized tools. If the indicators of loneliness are detected, practitioners should document them and facilitate follow-up evaluations and treatment accordingly. It is suggested that healthcare providers should actively schedule and promote activities that would promote interpersonal goals with more opportunities given to older adults with functional impairment. Future research could benefit from considering interpersonal goals’ effect on loneliness among older adults from varied cultural and religious backgrounds.

## 5. Conclusions

The findings of this study can help us to better understand the relationship between loneliness and interpersonal goals in older adults. Further, the findings highlight that interpersonal goals are significantly related to loneliness. Loneliness exhibits significant detrimental effects on individuals’ health. Healthcare practitioners who work with older adults should take measures to enhance their interpersonal goals, with special attention given to those with functional impairment. Further, mitigating loneliness among older adults is not only beneficial for their life satisfaction and well-being but may also provide a psychosocial resource to help them better confront the challenges of aging.

## Figures and Tables

**Table 1:** Varimax Rotated Factor Loadings for Compassionate and Self-Image Goals Items.

	Factor	
Item	1	2
Compassion for others	0.60	0.13
Supportive of others	0.75	0.23
Avoid being selfish	0.52	0.31
Avoid appearing unattractive	0.34	0.24
Get others to see your positive qualities	0.37	0.61
Get others to respect you	0.16	0.82

**Table 2:** Frequencies and Percentages for Categorical Variables.

Variable	Frequency	Percent
Age (years)		
65–74	1442	44.9
75–84	1358	42.3
≥85	412	12.9
Gender		
Male	1281	39.9
Female	1931	60.1
Ethnicity		
Not Hispanic	2909	90.6
Hispanic	303	9.4
Race		
White/Caucasian	2599	81.0
Black/African American	455	14.2
Other	155	4.8
Education		
Less than high school	488	15.2
GED	143	4.5
High-school graduate	995	31.0
Some college	790	24.6
College and above	795	24.8
Employment		
Employed	613	19.1
Unemployed	28	0.9
Retired or not in labor force	2571	80.0
Home health care		
No	2877	89.6
Yes	335	10.4
Functional impairment		
No	2683	83.5
Yes	529	16.5

**Table 3:** Complex Samples General Linear Model with Compassionate Goals Predicting Loneliness.

			95% CI B				
	B	*SE*	Lower	Upper	*t*	*df*	*p*-Value
(Intercept)	2.27	0.12	2.04	2.51	19.27	56	<0.001
Compassionate goals	−0.16	0.01	−0.19	−0.13	−11.93	56	<0.001
Age	0.00	0.00	−0.01	0.00	−1.08	56	0.286
Sex [Female]	0.01	0.02	−0.03	0.05	0.60	56	0.551
Race [White/Caucasian]	−0.03	0.04	−0.11	0.04	−0.95	56	0.348
Race [Black/African American]	−0.04	0.04	−0.13	0.04	−1.10	56	0.277
Race [Hispanic]	0.01	0.04	−0.06	0.09	0.35	56	0.730
Education [GED]	0.05	0.05	−0.05	0.14	0.97	56	0.336
Education [High school graduate]	−0.02	0.03	−0.08	0.04	−0.79	56	0.433
Education [Some college]	0.01	0.03	−0.05	0.07	0.27	56	0.792
Education [College and above]	−0.09	0.03	−0.14	−0.03	−3.11	56	0.003
Income	0.00	0.00	0.00	0.00	−2.95	56	0.005
Number of people in household	0.00	0.01	−0.02	0.02	−0.09	56	0.929
Employment [Employed]	−0.04	0.02	−0.09	0.01	−1.67	56	0.100
Employment [Unemployed]	0.19	0.12	−0.06	0.43	1.52	56	0.135
Functional impairment [Yes]	0.12	0.03	0.05	0.18	3.79	56	<0.001
Home health care [Yes]	0.08	0.03	0.02	0.13	2.59	56	0.012

**Table 4:** Complex Samples General Linear Model Predicting Loneliness (Full Model).

			95% CI B				
	B	*SE*	Lower	Upper	*t*	*df*	*p*-Value
(Intercept)	2.30	0.12	2.07	2.53	19.83	56	<0.001
Compassionate goals	−0.14	0.02	−0.17	−0.11	−9.19	56	<0.001
Self-image goals	−0.03	0.01	−0.06	−0.01	−2.50	56	0.015
Age	0.00	0.00	0.00	0.00	−1.00	56	0.320
Sex [Female]	0.01	0.02	−0.03	0.05	0.66	56	0.513
Race [White/Caucasian]	−0.03	0.04	−0.11	0.04	−0.94	56	0.352
Race [Black/African American]	−0.05	0.04	−0.13	0.03	−1.17	56	0.247
Race [Hispanic]	0.01	0.04	−0.07	0.09	0.29	56	0.776
Education [GED]	0.05	0.05	−0.05	0.14	0.99	56	0.328
Education [High school graduate]	−0.02	0.03	−0.08	0.04	−0.71	56	0.483
Education [Some college]	0.02	0.03	−0.05	0.08	0.48	56	0.635
Education [College and above]	−0.08	0.03	−0.13	−0.02	−2.85	56	0.006
Income	0.00	0.00	0.00	0.00	−2.92	56	0.005
Number of people in household	0.00	0.01	−0.02	0.02	−0.13	56	0.897
Employment [Employed]	−0.04	0.03	−0.09	0.01	−1.49	56	0.142
Employment [Unemployed]	0.19	0.12	−0.06	0.43	1.54	56	0.130
Functional impairment [Yes]	0.11	0.03	0.05	0.17	3.70	56	<0.001
Home health care [Yes]	0.08	0.03	0.02	0.14	2.72	56	0.009

**Table 5:** Complex Samples General Linear Model with Self-Image Goals Predicting Loneliness.

			95% CI B				
	B	*SE*	Lower	Upper	*t*	*df*	*p*-Value
(Intercept)	1.97	0.12	1.74	2.21	16.83	56	<0.001
Self-image goals	−0.09	0.01	−0.12	−0.07	−7.96	56	<0.001
Age	0.00	0.00	0.00	0.00	−0.62	56	0.538
Sex [Female]	−0.02	0.02	−0.06	0.02	−0.95	56	0.347
Race [White/Caucasian]	−0.04	0.04	−0.12	0.04	−1.01	56	0.317
Race [Black/African American]	−0.05	0.04	−0.14	0.03	−1.29	56	0.203
Race [Hispanic]	0.04	0.04	−0.04	0.12	0.96	56	0.342
Education [GED]	0.01	0.05	−0.09	0.10	0.13	56	0.895
Education [High school graduate]	−0.05	0.03	−0.11	0.01	−1.71	56	0.094
Education [Some college]	−0.02	0.03	−0.08	0.04	−0.67	56	0.506
Education [College and above]	−0.12	0.03	−0.17	−0.06	−4.29	56	<0.001
Income	0.00	0.00	0.00	0.00	−2.63	56	0.011
Number of people in household	0.00	0.01	−0.02	0.02	−0.11	56	0.911
Employment [Employed]	−0.04	0.03	−0.09	0.02	−1.33	56	0.188
Employment [Unemployed]	0.17	0.12	−0.07	0.41	1.45	56	0.152
Functional impairment [Yes]	0.11	0.03	0.05	0.17	3.78	56	<0.001
Home health care [Yes]	0.08	0.03	0.02	0.14	2.85	56	0.006

## Data Availability

The study data were derived from the 2016 HRS core dataset of the public biennial survey available at https://hrs.isr.umich.edu/about (accessed on 6 November 2020).
